# BATF2 prevents T-cell-mediated intestinal inflammation through regulation of the IL-23/IL-17 pathway

**DOI:** 10.1093/intimm/dxz014

**Published:** 2019-02-12

**Authors:** Hisako Kayama, Haruka Tani, Shoko Kitada, Anunya Opasawatchai, Ryu Okumura, Daisuke Motooka, Shota Nakamura, Kiyoshi Takeda

**Affiliations:** 1 Department of Microbiology and Immunology, Graduate School of Medicine, Osaka University, Osaka, Japan; 2 WPI Immunology Frontier Research Center, Osaka University, Osaka, Japan; 3 Core Research for Evolutional Science and Technology, Japan Agency for Medical Research and Development, Tokyo, Japan; 4 Genome Information Research Center, Research Institute for Microbial Diseases, Osaka University, Osaka, Japan; 5 Integrated Frontier Research for Medical Science Division, Institute for Open and Transdisciplinary Research Initiatives, Osaka University, Osaka, Japan; 6 Faculty of Dentistry, Mahidol University, Bangkok, Thailand

**Keywords:** colitis, gut homeostasis, IL-23/IL-17 pathway, ileitis, innate myeloid cell

## Abstract

Inappropriate activation of the IL-23 signaling pathway causes chronic inflammation through the induction of immunopathological T_h_17 cells in several tissues including the intestine, whereas adequate T_h_17 responses are essential for host defense against harmful organisms. In the intestinal lamina propria, IL-23 is primarily produced by innate myeloid cells including dendritic cells (DCs) and macrophages (Mϕs). However, the molecular mechanisms underlying the regulation of IL-23 production by these cells remains poorly understood. In this study, we demonstrated that BATF2 regulates intestinal homeostasis by inhibiting IL-23-driven T-cell responses. *Batf2* was highly expressed in intestinal innate myeloid subsets, such as monocytes, CD11b^+^ CD64^+^ Mϕs and CD103^+^ DCs. *Batf2*^−/−^ mice spontaneously developed colitis and ileitis with altered microbiota composition. In this context, IL-23, but not TNF-α and IL-10, was produced in high quantities by intestinal CD11b^+^ CD64^+^ Mϕs from *Batf2*^−/−^ mice compared with wild-type mice. Moreover, increased numbers of IFN-γ^+^, IL-17^+^ and IFN-γ^+^ IL-17^+^ CD4^+^ T cells, but not IL-10^+^ CD4^+^ T cells, accumulated in the colons and small intestines of *Batf2*^−/−^ mice. In addition, RORγt-expressing innate lymphoid cells were increased in *Batf2*^−/−^ mice. *Batf2*^−/−^*Rag2*^−/−^ mice showed a reduction in intestinal inflammation present in *Batf2*^−/−^ mice. Furthermore, the high numbers of intestinal IL-17^+^ and IFN-γ^+^ IL-17^+^ CD4^+^ T cells were markedly reduced in *Batf2*^−/−^ mice when introducing *Il23a* deficiency, which was associated with the abrogation of intestinal inflammation. These results indicated that BATF2 in innate myeloid cells is a key molecule for the suppression of IL-23/IL-17 pathway-mediated adaptive intestinal pathology.

## Teaser

BATF2 prevents colitis by regulating macrophage IL-23 production

## Introduction

Inflammatory bowel diseases (IBDs), which include Crohn’s disease (CD) and ulcerative colitis (UC), are characterized by chronic and relapsing-remitting intestinal inflammation (1). The incidence and prevalence of IBD are increasing globally (2, 3), but the etiology of IBD remains poorly understood. An excess of adaptive immune responses driven by pro-inflammatory cytokine IFN-γ-producing CD4^+^ T (T_h_1) cells and IL-17-producing CD4^+^ T (T_h_17) cells was reported in the intestinal lamina propria of patients with IBD (4). Both T_h_1 and T_h_17 responses function in host defense against invading pathogens, while inadequate effector responses lead to damage of the intestinal mucosa. Because innate myeloid cells instruct the adaptive immune system through antigen presentation and production of anti- and pro-inflammatory cytokines via activation of the pattern recognition receptors signaling pathway (4–6), dysregulation of innate immune responses is implicated in the pathogenesis of intestinal inflammation. IBD patients have increased numbers of intestinal macrophages (Mϕs) with elevated production of pro-inflammatory cytokines in response to gut microbiota and enhanced antigen presentation (7, 8). To maintain intestinal tolerance, the development (9–12) and activation (13–15) of intestinal Mϕs are strictly regulated under the homeostatic conditions.

IL-23, a pro-inflammatory cytokine formed by a heterodimer of the IL-23p19 and IL-12p40 subunits, maintains developing T_h_17 cells (5, 16). Genome-wide association studies (GWAS) indicated that polymorphisms of IL-23/T_h_17 axis-related genes including *IL23R*, *STAT3* and *IL21* are IBD risk factors (17–20). Accordingly, murine studies demonstrated that the IL-23/IL-23 receptor (IL-23R) signaling pathway exacerbated intestinal inflammation by activating innate immune cells (21–25) or T cells (26–28). The beneficial effects of IL-23 during pathogenic bacterial infection in the intestine have also been reported (29–31). In the intestine, IL-23 production is negatively regulated in innate myeloid cells via IL-10R-dependent signaling (14, 15, 32). However, the mechanism underlying the modulation of IL-23 production in intestinal innate myeloid cells is largely unknown.

The transcription factor BATF2, which belongs to the BATF family, was initially characterized as an inhibitor of tumor growth through the suppression of AP-1 activity (33). In addition, BATF2 prevented colonic tumorigenesis and angiogenesis by negatively regulating the HIF-1α/VEGF axis (34). Recent studies have shown that BATF2 is important for appropriate innate immune responses. In Mϕs infected with *Mycobacterium tuberculosis*, BATF2 induced the expression of inflammatory mediators including *Tnf*, *Il12b*, *Nos2* and *Ccl5*, by interacting with IRF1 *in vitro* (35). In tumor-associated Mϕs, the expression of *Il12b* was facilitated through interactions between BATF2 and the p65/p50 heterodimer, leading to the induction of anti-tumor adaptive immune responses (36). We previously reported that BATF2 down-regulated the expression of *Il23a* by binding directly to c-JUN in *Trypanosoma cruzi*-infected Mϕs and dendritic cells (DCs), thereby suppressing immunopathological T_h_17 responses (37). However, whether BATF2 exerts its immunoregulatory effect under homeostatic conditions remains unknown.

In this study, we analyzed the function of BATF2 in the maintenance of gut homeostasis. *Batf2* deficiency in mice resulted in the development of spontaneous colitis and ileitis. BATF2 negatively regulated the production of IL-23 by CD11b^+^ CD64^+^ Mϕs, subsequently suppressing IL-17-producing CD4^+^ T-cell-induced intestinal pathology. Therefore, the BATF2-mediated regulation of the IL-23/IL-17 axis is required for the prevention of T-cell-mediated intestinal inflammation.

## Methods

### Mice

C57BL/6J mice were purchased from Japan SLC (Hamamatsu, Japan). *Batf2*^−/−^ and *Il23a*^−/−^ mice were generated as described previously (37). *Batf2*^−/−^ mice were co-housed with their wild-type littermates up to 6 weeks of age and subsequently housed single up to the age of 40 weeks. All mice were maintained under specific pathogen-free conditions. All animal experiments were conducted in accordance with the guidelines of the Animal Care and Use Committee of Osaka University.

### Reagents

Dextran sulfate sodium salt was purchased from MP Biomedicals (Kaysersberg, France). The Ca^2+^ ionophore A23187, phorbol myristate acetate (PMA) and lipopolysaccharide (LPS; O55: B5) were purchased from Sigma-Aldrich (St Louis, MO, USA). The transcription Factor Buffer Set was purchased from BD Pharmingen (Franklin Lakes, NJ, USA).

### Flow cytometry

The following antibodies were purchased from BD Biosciences (Franklin Lakes, NJ, USA): 7-AAD, Fixable Viability Stain 510, anti-mouse CD16/32 (clone 2.4.G2), PE-Cy7-conjugated anti-mouse-Ly-6C (clone AL-21), Pacific blue-conjugated anti-mouse CD45R (RA3-6B2), PE-conjugated anti-mouse CD64 (X54-5/7.1), FITC-conjugated anti-mouse Ly6G (1A8), APC-conjugated anti-mouse-CD11c (clone HL3), BV510-conjugated anti-mouse CD3e (145-2C11), BV510-conjugated anti-mouse CD19 (1D3) and BV421-conjugated anti-mouse RORγt (Q31-378). Pacific blue-conjugated anti-mouse CD11b (clone M1/70), Pacific blue-conjugated anti-mouse CD8a (53-6.7), Percp-Cy5.5- or APC/Cy7-conjugated anti-mouse CD4 (clone CK1.5), APC-conjugated anti-mouse IL-17A (clone TC11-18H10.1), FITC-conjugated anti-mouse IFN-γ (clone XMG1.2), PE-conjugated anti-mouse IL-10 (clone JES5-16E3), FITC-conjugated anti-mouse CD19 (6D5), PE/Cy7-conjugated anti-mouse CD127 (A7R34), APC-conjugated anti-mouse CD196 (29-2L17), FITC-conjugated anti-mouse CD335 (29A1.4), Pacific blue-conjugated anti-mouse CD90.2 (53-2.1), APC-conjugated anti-mouse IL-22 (Poly5164), and PerCP-, FITC- or PE/Cy7-conjugated anti-mouse CD45 (clone 30-F11) antibodies were purchased from BioLegend (San Diego, CA, USA). APC-conjugated anti-mouse Foxp3 antibody (clone 3G3) and FITC-conjugated anti-mouse CD3e (145-2C11) were purchased from TONBO Biosciences (Tucson, AZ, USA). PE-conjugated anti-mouse IgA (mA-6E1) and FITC-conjugated anti-mouse MHCII (M5/14.15.2) were purchased from eBioscience (San Diego, CA, USA). Flow cytometric analysis was performed with a FACSCanto II flow cytometer (BD Biosciences) with FlowJo software (Tree Star, Ashland, OR, USA). Murine large intestinal cells were isolated with a FACSAria flow cytometer (BD Biosciences). The instrumental compensation was set in each experiment using two-color, three-color, four-color and six-color stained samples.

### Quantitative RT–PCR

Total RNA was isolated using the RNeasy Mini Kit (Qiagen, Manchester, UK), and the RNA was reverse transcribed with Moloney Murine Leukemia Virus Reverse Transcriptase (M-MLV) (Promega, Madison, WI, USA) and random primers (Toyobo, Tokyo, Japan) after treatment with RQ1 DNase I (Promega). Quantitative RT–PCR was performed on a Step One Plus™ Real-Time PCR System (Applied Biosystems) using GoTaq qPCR Master Mix (Promega). All values were normalized to the expression of *Gapdh*, encoding glyceraldehyde-3-phosphate dehydrogenase, and the fold difference in expression relative to that of *Gapdh* is shown. The previously described primer sets for *Gapdh*, *Il23a*, *Tnf*, *Il10* and *Batf2* were used (37). The amplification conditions were 50°C (2 min), 95°C (10 min) and 40 cycles of 95°C (15 s) and 60°C (60 s).

### Isolation of immune cells from the intestine

Murine innate myeloid cells and lymphocytes were isolated from mouse intestines (38) as described previously. Cell surface/intracellular staining of intestinal CD4^+^ T cells stimulated with 50 ng ml^−1^ PMA and 5 μM calcium ionophore A23187 in complete RPMI 1640 at 37 °C for 4 h in the presence of GolgiStop was performed with a Cytofix/Cytoperm Plus Kit (BD Biosciences) in accordance with the manufacturer’s instructions. Foxp3 expression in CD4^+^ T cells isolated from the large intestine was analyzed with a Foxp3/Transcription Factor Staining Buffer set (eBioscience). Large intestinal innate lymphoid cells (ILCs) were isolated as previously described (39) and cell surface/intracellular staining was performed as previously described (40).

### Cytokine analysis

The concentrations of TNF-α, IL-10, IL-23, IL-17A and IFN-γ in culture supernatants were measured with a Cytometric Bead Array (CBA) kit (BD Biosciences).

### Histopathological analysis

Large intestines and ileums collected from wild-type, *Rag2*^−/−^, *Il23a*^−/−^, *Batf2*^−/−^, *Batf2*^−/−^*Rag2*^−/−^ and *Batf2*^−/−^*Il23a*^−/−^ mice aged older than 28 weeks were fixed in 4% paraformaldehyde. Paraffin-embedded 5-μm sections mounted on glass slides were used for hematoxylin and eosin (H&E) staining, and tissue histopathology was examined by light microcopy. Each section of large intestines was evaluated for inflammation scores as previously described (41).

### Determination of microbiota by deep sequencing

Extraction of bacterial DNA from feces was performed as described previously (42). Libraries were prepared in accordance with the ‘Illumina 16S Metagenomic Sequencing Library Preparation Guide’ with a primer set (27Fmod: 5′-AGRGTTTGATCMTGGCTCAG-3′ and 338R: 5′-TGCTGCCTCCCGTAGGAGT-3′) targeting the V1–V2 region of the 16S rRNA gene. Then, 251 bp paired-end sequencing of the amplicon was performed on a MiSeq (Illumina) using a MiSeq v2 500 cycle kit. Raw paired-end sequences were merged using PEAR (http://sco.h-its.org/exelixis/web/software/pear/), and 50 000 reads per sample were randomly selected using seqtk (https://github.com/lh3/seqtk) for further analysis. The processed sequences were clustered into operational taxonomic unit (OTU) defined at a 97% similarity cutoff using UCLUST version 1.2.22q. Representative sequences for each OTU were then classified taxonomically using RDP Classifier version 2.2, with the Greengenes 13_8 database. The bioinformatics pipeline QIIME, version 1.9.1, was used as the informatics environment for all relevant processing of sequencing data and calculation of relative bacterial abundances.

### Statistical analysis

Differences between the control and experimental groups were evaluated by Student’s *t*-test. Differences where *P* <0.05 were considered statistically significant.

## Results

### Innate myeloid cell-specific expression of Batf2 in the large intestine

**Fig. 1.**
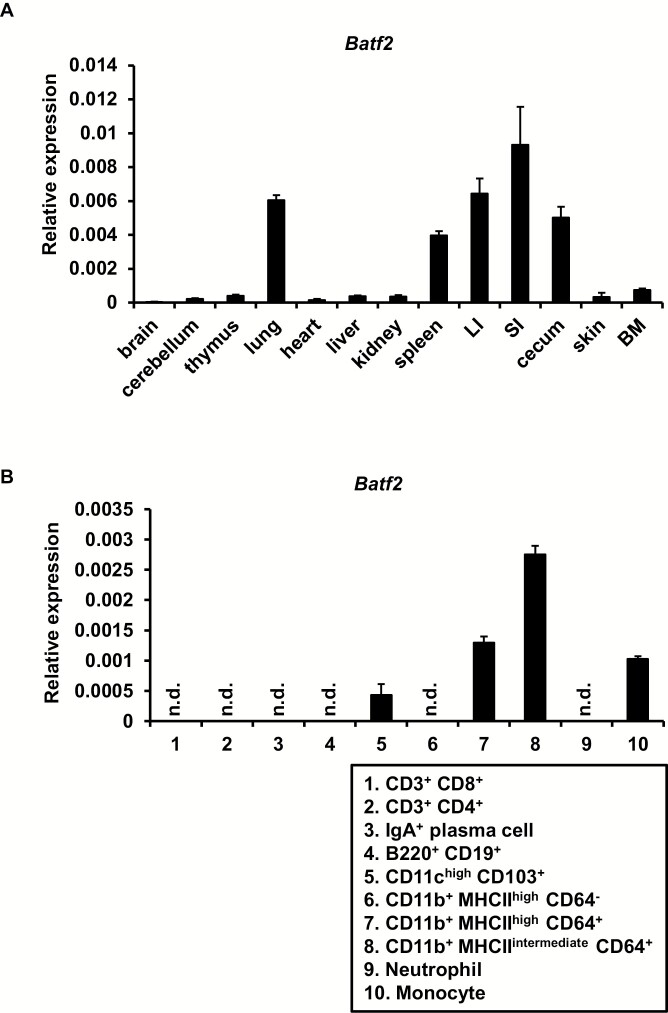
*Batf2* is highly expressed in several innate myeloid subsets of the large intestine. (A) Quantitative RT–PCR analysis of the mRNA expression of *Batf2* in various organs. Data are representative of two independent experiments. Graphs show the mean ± SD. BM, bone marrow; LI, large intestine; SI, small intestine. (B) Quantitative RT–PCR analysis of the mRNA expression of *Batf2* in the indicated cell populations from the colons of C57BL/6J mice. Data are representative of two independent experiments. Graphs show the mean ± SD. n.d., not detected.

To determine the role of BATF2 under the steady state, we analyzed tissue expression of *Batf2*. Higher expression of *Batf2* was observed in the spleen, lung, small intestine, cecum and large intestine of wild-type mice (Fig. 1A). We previously demonstrated that *Batf2* was expressed in CD11b^+^ F4/80^+^ macrophages (Mϕs) in the spleen, but not adaptive lymphoid cells, and that it contributed to the suppression of immunopathological T_h_17 responses during *T. cruzi* infection (37). A recent study clearly demonstrated that *Batf2* expression induced in Mϕs in the lung during infection with *M. tuberculosis* and *Listeria monocytogenes* was associated with the pathogenesis of type 1 infectious diseases (43). In addition, this study showed that BATF2 contributed to prevention of type 2 infectious disease caused by *Schistosoma mansoni* infection in the small intestine. However, the role of BATF2 in the large intestine remains unknown. Therefore, we next attempted to identify *Batf2*-expressing cell subsets in the large intestine. In the lamina propria of the large intestine, innate myeloid cells, such as CD11c^high^ CD103^+^ DCs, CD11b^+^ CD64^+^ Mϕs and CD11b^+^ Ly6C^+^ monocytes, expressed *Batf2*, whereas CD11b^+^ Ly6G^+^ neutrophils and CD11b^+^ MHC II^high^ CD64^−^ DCs did not (Fig. 1B). In accordance with *Batf2* expression profiles in the spleen, adaptive immune cells, including T cells, plasma cells and B cells, did not express *Batf2* in the large intestine. Thus, *Batf2* is highly expressed in some subsets of innate myeloid cells residing in the large intestine as well as the lung and spleen.

### BATF2 deficiency induces the development of spontaneous intestinal inflammation with altered microbiota composition

**Fig. 2.**
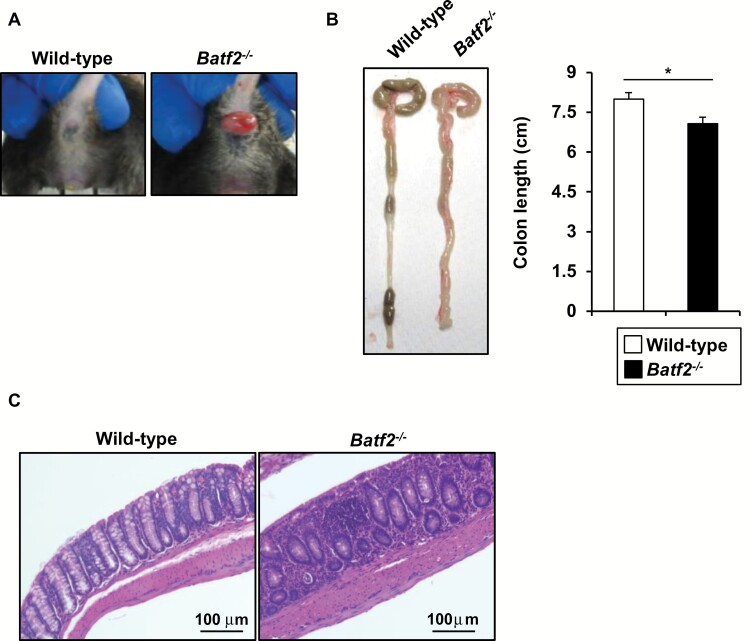
*Batf2* deficiency induces spontaneous colitis. (A) Data are representative of the rectal prolapse of wild-type and *Batf2*^−/−^ mice. (B) Colon length of over 28-week-old wild-type (*n* = 7) and *Batf2*^−/−^ (*n* = 7) mice. *P < 0.05. All graphs show the mean ± SEM. (C) Representative colon sections of 28-week-old wild-type and *Batf2*^−/−^ mice.

**Fig. 3.**
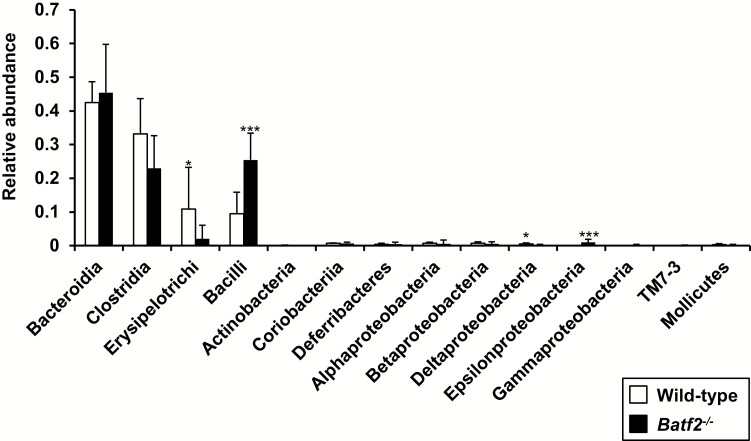
Altered composition of fecal microbiota in wild-type and *Batf2*^−/−^ mice. The mean abundance of the listed commensal classes were determined by DNA sequencing of *16S* ribosomal DNA in feces from single housed 12-week-old wild-type (*n* = 9) and *Batf2*^−/−^ (*n* = 10) mice (mean values ± SD). *P < 0.05, ***P < 0.005. n.s., not significant.

While analyzing the physiological role of BATF2 during *T. cruzi* infection (37), we found that *Batf2*^−/−^ mice, but not littermate wild-type mice, developed rectal prolapse over at the age of 28 weeks (Fig. 2A). In addition, almost all of *Batf2*^−/−^ mice exhibited colon shortening and severe large intestinal pathology with an increased number of inflammatory cells (Fig. 2B and C). Moreover, the infiltration of inflammatory cells was increased in the ileum of *Batf2*^−/−^ mice (Supplementary Figure 1A), indicating that the lack of BATF2 resulted in the development of spontaneous colitis and ileitis. The altered composition of microbiota, termed dysbiosis, was reported in several animal models of spontaneous colitis including *Muc2*^−/−^ (44), *Mdr1a*^−/−^ (45), *Il10*^−/−^ (46) and TRUC (47) mice. Therefore, we analyzed the bacterial composition of the feces from wild-type and *Batf2*^−/−^ mice by 16S rRNA gene deep sequencing. Class-level fecal bacterial composition was altered between wild-type and *Batf2*^−/−^ mice at 8 weeks after starting single-housing (Fig. 3). The relative abundance of *Bacilli* and *Epsilonproteobacteria* in *Batf2*^−/−^ mice was higher than that in wild-type mice, whereas *Erysipelotrichia* and *Deltaproteobacteria* were less abundant in *Batf2*^−/−^ mice. Thus, BATF2 is required for the inhibition of spontaneous colitis accompanied with dysbiosis.

### BATF2 in intestinal CD11b^+^ CD64^+^ Mϕs negatively regulates the production of IL-23, but not TNF-α and IL-10

**Fig. 4.**
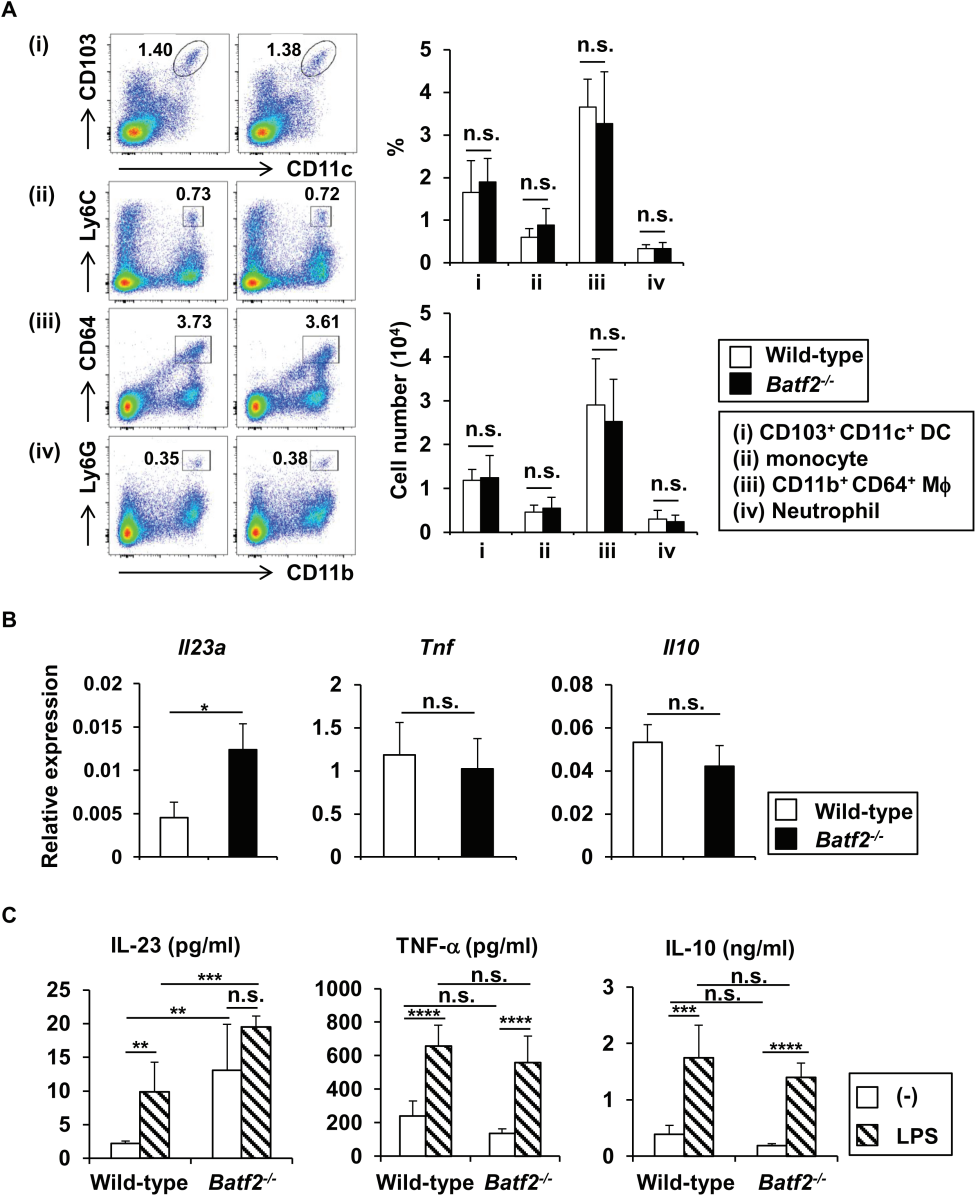
BATF2 contributes to the suppression of IL-23 production by CD11b^+^ CD64^+^ Mϕs in the colon. (A) Flow cytometric plot (left panel), frequency (right upper panel) and number (right bottom panel) of CD103^+^ CD11c^+^ (i), CD11b^+^ Ly6C^+^ monocytes (ii), CD11b^+^ CD64^+^ macrophages (iii) and CD11b^+^ Ly6G^+^ neutrophils (iv) in the colons of 8-week-old wild-type (*n* = 5) and *Batf2*^−/−^ (*n* = 6) mice. (B) CD11b^+^ CD64^+^ macrophages from the colons of wild-type and *Batf2*^−/−^ mice were stimulated with LPS for 3 h and the expression levels of *Il23a*, *Tnf* and *Il10* were analyzed. Graphs show the mean ± SEM from four independent experiments. *P < 0.05. (C) Production of IL-23, TNF-α and IL-10 by large intestinal CD11b^+^ CD64^+^ Mϕs from 8-week-old wild-type and *Batf2*^−/−^ mice in the presence or absence of LPS. Graphs show the mean ± SEM from four independent experiments. **P < 0.01, ***P < 0.005, ****P < 0.001. n.s., not significant.

Because *Batf2*^−/−^ mice suffered from chronic intestinal inflammation, we attempted to elucidate the mechanism by which BATF2 modulates intestinal homeostasis. We first analyzed the composition of intestinal innate myeloid subsets (Fig. 4A). To exclude the possibility of inflammation-related changes in composition, we used 8-week-old *Batf2*^−/−^ mice, which showed no sign of intestinal pathology at the age. The frequencies and total numbers of CD103^+^ DCs, CD11b^+^ CD64^+^ Mϕs, monocytes and neutrophils were not altered in the large intestines of *Batf2*^−/−^ mice, indicating that BATF2 does not affect the development of intestinal innate myeloid cells. We previously showed that IFN-γ-inducible BATF2 in Mϕs during *T. cruzi* infection suppressed the production of IL-23 (37). Therefore, we next examined the expression of cytokine genes in CD11b^+^ CD64^+^ Mϕs from the large intestines of wild-type and *Batf2*^−/−^ mice at the age of 8 weeks (Fig. 4B). In LPS-stimulated CD11b^+^ CD64^+^ Mϕs from *Batf2*^−/−^ mice, the expression of *Il23a*, encoding IL-23p19, was higher compared with that in wild-type mice, despite the normal expression of *Tnf* and *Il10*. Because the enhanced expression of *Il23a* in large intestinal CD11b^+^ CD64^+^ Mϕs from *Batf2*^−/−^ mice was observed, we further assessed the production of IL-23, TNF-α and IL-10 by CD11b^+^ CD64^+^ Mϕs in the presence or absence of LPS (Fig. 4C). Without LPS stimulation, CD11b^+^ CD64^+^ Mϕs from the colons of *Batf2*^−/−^ mice produced higher amounts of IL-23 than by wild-type cells. In wild-type CD11b^+^ CD64^+^ Mϕs, IL-23 production was up-regulated in response to LPS, whereas a partial, but not significant, elevation of IL-23 production was observed in *Batf2*^−/−^ cells. There were no differences in the production levels of TNF-α and IL-10 by wild-type and *Batf2*^−/−^ CD11b^+^ CD64^+^ Mϕs stimulated with or without LPS. Thus, BATF2 is responsible for the down-regulation of IL-23 production by innate myeloid cells in the large intestine.

### Perturbation of adaptive immune responses is implicated in the pathogenesis of intestinal inflammation in *Batf2*^−/−^ mice

**Fig. 5.**
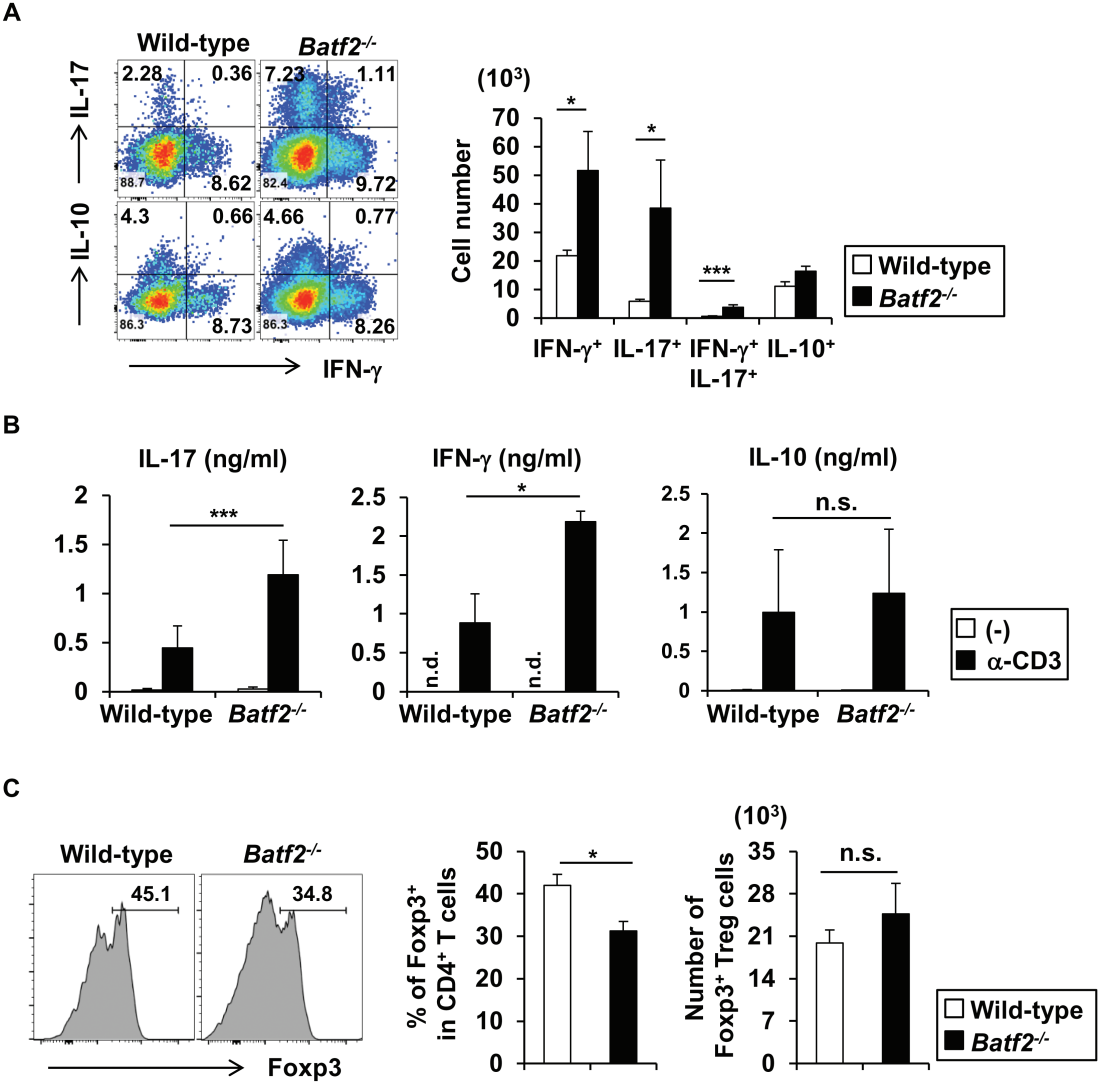
Infiltration of colitogenic effector CD4^+^ T cells is facilitated in the large intestinal lamina propria of *Batf2*^−/−^ mice. (A) Representative flow cytometric plots (left panel) and numbers (right panel) of IFN-γ-, IL-17- and IL-10-producing CD4^+^ T cells from the large intestine of 24-week-old wild-type (*n* = 4) and *Batf2*^−/−^ (*n* = 5) mice (mean values ± SEM). *P < 0.05, ***P < 0.005. Graphs represent data pooled from two independent experiments. (B) CD4^+^ T cells were isolated from the large intestines of 24-week-old wild-type and *Batf2^–^/^–^* mice. Isolated CD4^+^ T cells were stimulated with or without anti-CD3 antibody and analyzed for the production of IFN-γ, IL-17 and IL-10. Graphs show the mean values ± SEM from four independent experiments. *P < 0.05, ***P < 0.005. n.d., not detected. (C) Histogram (left panel), frequency (middle panel) and number (right panel) of Foxp3-expressing cells in CD4^+^ T cells from the large intestines of 24-week-old wild-type (*n* = 6) and *Batf2*^−/−^ (*n* = 6) mice. Graphs show the mean values ± SEM and data pooled from two independent experiments. *P < 0.05. n.s., not significant.

**Fig. 6.**
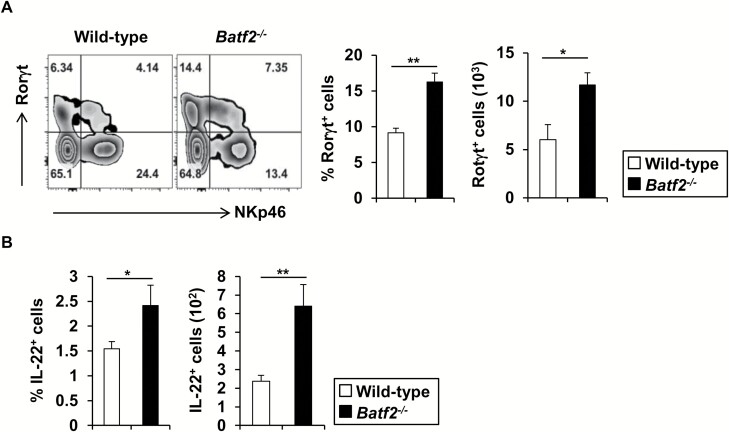
ILCs are increased in the large intestinal lamina propria of *Batf2*^−/−^ mice. (A) Representative zebra plots of RORγt- and NKp46-expressing Lin^−^ CCR6^−^ CD127^+^ CD45^+^ ILCs isolated from the large intestinal lamina propria of wild-type and *Batf2*^−/−^ mice (left panel). Frequency (middle panel) and number (right panel) of RORγt-expressing ILCs from the large intestines of 24-week-old wild-type (*n* = 6) and *Batf2*^−/−^ (*n* = 9) mice (mean values ± SEM). *P < 0.05, **P < 0.01. Graphs represent data pooled from two independent experiments. (B) Frequency (left panel) and number (right panel) of IL-22-producing ILCs from the large intestines of 24-week-old wild-type (*n* = 10) and *Batf2*^−/−^ (*n* = 14) mice. All graphs show the mean values ± SEM and data pooled from three independent experiments. *P < 0.05, **P < 0.01.

Several studies demonstrated that IL-23 contributed to T-cell-mediated intestinal pathology by driving the proliferation of T cells, accumulation of IL-17^+^ CD4^+^ T cells and induction of IFN-γ^+^ IL-17^+^ CD4^+^ T cells (26–28). Therefore, we analyzed CD4^+^ T-cell populations in the large intestinal lamina propria by intracellular staining (Fig. 5A). In *Batf2*^−/−^ mice, the numbers of IFN-γ^+^, IL-17^+^ and IFN-γ^+^ IL-17^+^ CD4^+^ T cells, but not IL-10^+^ CD4^+^ T cells, were markedly increased relative to those of wild-type mice. Consistent with the development of ileitis, elevated numbers of effector CD4^+^ T cells including IFN-γ^+^, IL-17^+^ and IFN-γ^+^ IL-17^+^ CD4^+^ T cells were present in the lamina propria of the small intestines of *Batf2*^−/−^ mice compared with wild-type mice, but no changes in the number of IL-10^+^ CD4^+^ T cells were observed (Supplementary Figure 1B). We measured the amounts of IFN-γ, IL-17 and IL-10 in culture supernatants of CD4^+^ T cells simulated with or without anti-CD3 antibody (Fig. 5B). In accordance with the intracellular staining analysis, the anti-CD3 antibody-induced secretion of IL-17 and IFN-γ by CD4^+^ T cells from the colons of *Batf2*^−/−^ mice was increased compared with wild-type mice, whereas the amount of IL-10 secretion was unaffected. A previous study showed that IL-23 signaling through IL-23R on intestinal T cells inhibited Foxp3^+^ regulatory T (T_reg_) cell differentiation, thereby exerting colitogenic activity (28). Although the robust production of IL-23 by CD11b^+^ CD64^+^ Mϕs was observed, the number of Foxp3^+^ T_reg_ cells in the large intestinal lamina propria of *Batf2*^−/−^ mice was similar to that of wild-type mice (Fig. 5C). These results suggest that BATF2 is required for the suppression of effector T-cell responses via a Foxp3^+^ T_reg_-cell-independent mechanism. In addition to CD4^+^ T cells, IL-23 can activate immunopathological RORγt^+^ innate lymphoid cells (group 3 ILCs: ILC3), which robustly produce T_h_17-related cytokines including IL-17, IL-22 and GM-CSF (22–25). Compared with wild-type mice, *Batf2*^−/−^ mice showed an elevated frequency and total cell number of large intestinal RORγt^+^ ILCs (Fig. 6A). In addition, an increase in IL-22-producing ILCs was observed in the colons of *Batf2*^−/−^ mice compared with wild-type mice (Fig. 6B), indicating that BATF2 is involved in the attenuation of ILC3 responses.

**Fig. 7.**
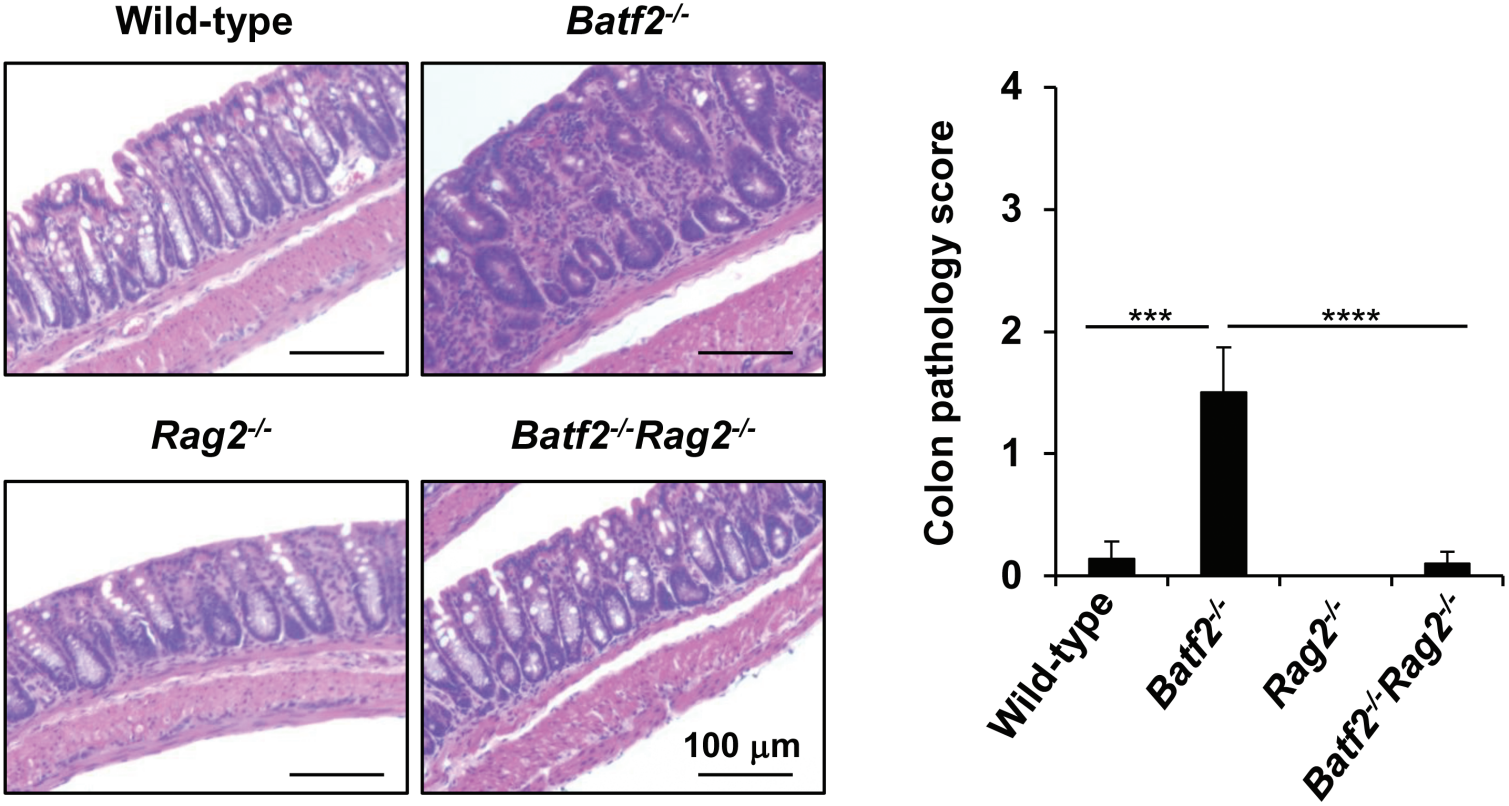
Adaptive lymphoid cells, but not ILCs, are involved in the pathogenesis of large intestinal inflammation in *Batf2*^−/−^ mice. Representative colon sections (left panel) and histopathology scores for the large intestines (right panel) 32- to 40-week-old wild-type (*n* = 7), *Batf2*^−/−^ (*n* = 10), *Rag2*^−/−^ (*n* = 5) and *Batf2*^−/−^*Rag2*^−/−^ (*n* = 10) mice. ***P < 0.005, ****P < 0.001. Graphs show the mean values ± SD.

To determine whether BATF2 limits innate intestinal pathology or adaptive intestinal pathology in the colon, we generated *Batf2*^−/−^*Rag2*^−/−^ mice. Compared to wild-type mice, *Batf2*^−/−^ mice exhibited worsened colonic histopathology (Fig. 7), which was identified by quantifying the degree of goblet cells loss, mucosa thickening, infiltration of inflammatory cells, ulcers and crypt abscess (41). However, the absence of adaptive immune cells induced by the lack of *Rag2* in *Batf2*^−/−^ mice led to the suppression of spontaneous intestinal inflammation. These findings demonstrated that BATF2 is required for the prevention of spontaneous colitis caused by disrupted adaptive immune responses.

### Lack of IL-23 production suppresses the development of IL-17-producing CD4^+^ T-cell-mediated colitis in *Batf2*^−/−^ mice

**Fig. 8.**
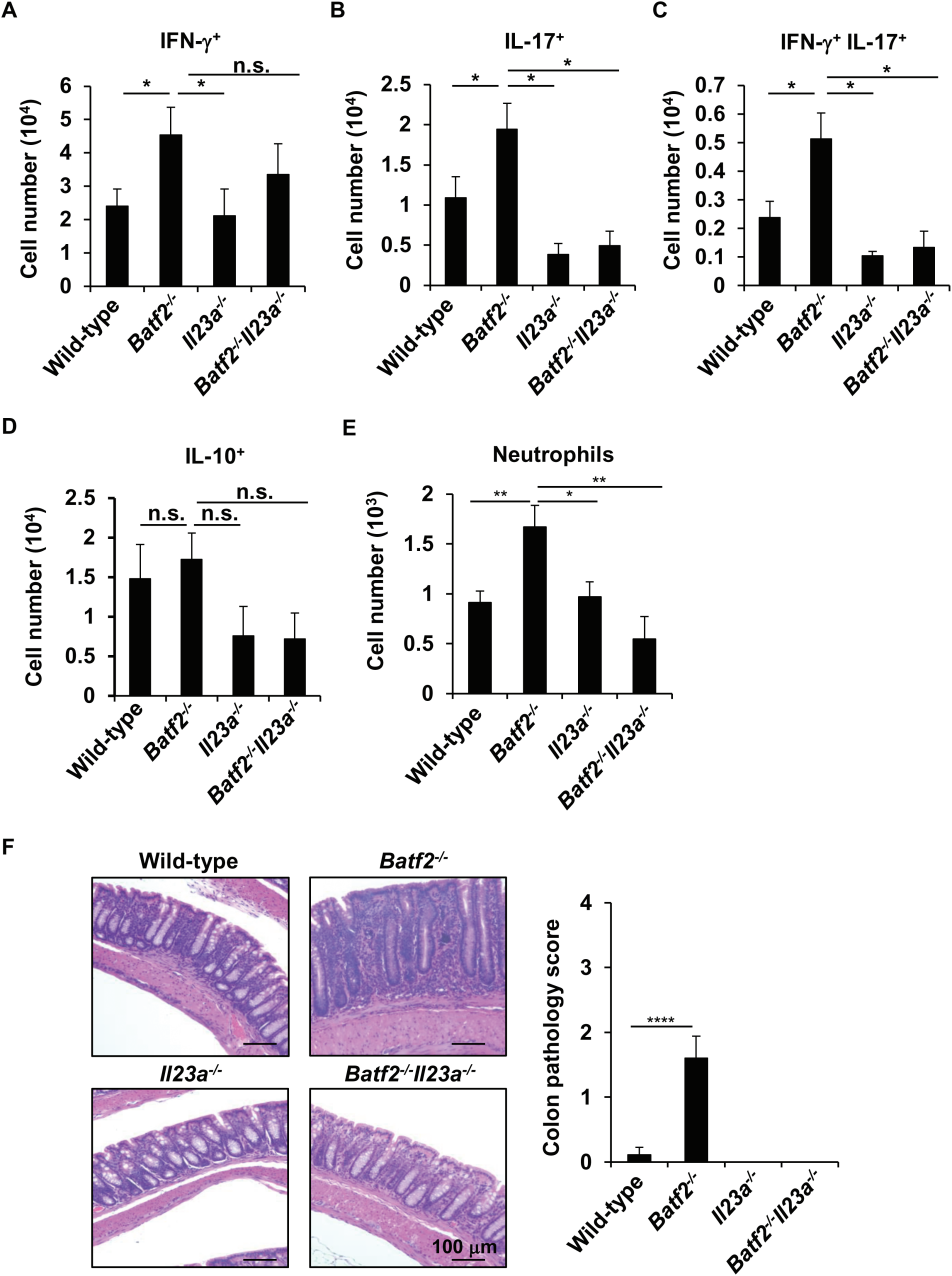
*Il23a* deficiency suppresses spontaneous colitis in *Batf2*^−/−^ mice by reducing colitogenic effector CD4^+^ T cells. (A–D) Numbers of IFN-γ- (A), IL-17- (B), IFN-γ and IL-17- (C) and IL-10 (D)-producing CD4^+^ T cells in the large intestines of 28-week-old wild-type (*n* = 10), *Batf2*^−/−^ (*n* = 12), *Il23a*^−/−^ (*n* = 4) and *Batf2*^−/−^*Il23a*^−/−^ (*n* = 4) mice (mean values ± SD). Graphs represent data pooled from two independent experiments. *P < 0.05. n.s., not significant. (E) Number of CD11b^+^ Ly6G^+^ neutrophils in the large intestines of 24- or 28-week-old wild-type (*n* = 9), *Batf2*^−/−^ (*n* = 12), *Il23a*^−/−^ (*n* = 5) and *Batf2*^−/−^*Il23a*^−/−^ (*n* = 4) mice. All graphs show the mean values ± SEM and data pooled from two independent experiments. *P < 0.05, **P < 0.01. (F) Representative colon sections (left panel) and histopathological scores (right panel) of 40-week-old wild-type (*n* = 8), *Batf2*^−/−^ (*n* = 10), *Il23a*^−/−^ (*n* = 5) and *Batf2*^−/−^*Il23a*^−/−^ (*n* = 6) mice. Graphs show the mean values ± SD. ****P < 0.001.

To investigate whether high levels of IL-23 production are involved in the intestinal inflammation in *Batf2*^−/−^ mice, we analyzed *Batf2*^−/−^*Il23a*^−/−^ mice. In the large intestinal lamina propria of wild-type, *Batf2*^−/−^, *Il23a*^−/−^ and *Batf2*^−/−^*Il23a*^−/−^ mice, the numbers of IFN-γ^+^, IL-17^+^, IFN-γ^+^ IL-17^+^ and IL-10^+^ CD4^+^ T cells were evaluated (Fig. 8A–D). There was no difference in the number of IL-10^+^ CD4^+^ T cells in the colons between the four groups. The introduction of *Il23a* deficiency into *Batf2*^−/−^ mice resulted in a marked reduction of IL-17^+^ CD4^+^ T cells and IL-17^+^ IFN-γ^+^ CD4^+^ T cells in the large intestines, while partially increased numbers of IFN-γ^+^ CD4^+^ T cells were observed in *Batf2*^−/−^*Il23a*^−/−^ mice. In accordance with the decreased number of IL-17-producing CD4^+^ T cells, a low number of infiltrating neutrophils was observed in the lamina propria of the large intestines of *Batf2*^−/−^*Il23a*^−/−^ mice (Fig. 8E). In addition, *Batf2*^−/−^*Il23a*^−/−^ mice showed attenuated large intestinal inflammation, as determined by lower pathological scores (Fig. 8F). These results indicate that the augmented production of IL-23 by innate myeloid cells is associated with the accumulation of IL-17-producing CD4^+^ T cells, which is implicated in the pathogenesis of intestinal inflammation in *Batf2*^−/−^ mice.

## Discussion

In this study, we identified the mechanism underlying the suppression of IL-23/IL-17 pathway-mediated intestinal inflammation by BATF2 under steady state conditions. In the intestinal mucosa, BATF2, which is constitutively expressed in innate myeloid cells, such as CD11b^+^ CD64^+^ Mϕs, is required to inhibit IL-23 production, leading to regulation of the IL-23/IL-17 axis that is associated with the pathogenesis of mucosal inflammation in the intestine.

During *T. cruzi* infection, *Batf2* expression is induced in splenic Mϕs and bone marrow-derived Mϕs by an IFN-γ-dependent mechanism (37). In the intestine, the expression of *Batf2* in CD11b^+^ CD64^+^ Mϕs from the colons of *Ifng*^−/−^ mice (*Batf2*/*Gapdh*: 0.0014 ± 0.00005: our unpublished results) was partially decreased compared with wild-type mice (*Batf2*/*Gapdh*: 0.0027 ± 0.0001: our unpublished results). A previous study showed that type I IFNs induced the expression of *Batf2* in tumor cells (33). Several functions of type I IFNs in the maintenance of gut homeostasis or pathogenesis of intestinal inflammation have been reported (48). Interestingly, the locus harboring IFNAR, a receptor for type I IFNs, was reported as a susceptibility region for IBD (48). Therefore, it would be interesting to investigate whether type I IFNs induce the expression of *Batf2* through IFNAR on intestinal myeloid cells. In addition to Mϕs, we observed the constitutive expression of *Batf2* in CD103^+^ DCs from the intestinal lamina propria. These cells produce IL-23 in response to flagellin (30). Thus, BATF2 might regulate the Toll-like receptor 5 (TLR5)-induced expression of *Il23a* in intestinal CD103^+^ DCs.

In the current study, we showed that CD11b^+^ CD64^+^ Mϕs from the colon of *Batf2*^−/−^ mice produced higher amounts of IL-23 than wild-type cells in the presence or absence of LPS. However, IL-23 production by *Batf2*^−/−^ CD11b^+^ CD64^+^ Mϕs did not increase after LPS stimulation, while production of IL-10 and TNF-α was markedly increased. A previous study demonstrated that autocrine IL-10 signaling in intestinal Mϕs suppresses their IL-23 production during *Citrobacter rodentium* infection and thereby prevents intestinal pathology (32). In addition, Mϕs from the colon of *Il10ra*-deficient mice that spontaneously develop colitis exhibited elevated TLR-induced expression of *Il23a*, but not *Il6* and *Tnf* (15). Therefore, colitogenic IL-23 production by intestinal Mϕs via TLR signaling might be tightly regulated in at least two ways including a BATF2-dependent mechanism and an IL-10-dependent mechanism and disruption of either of the mechanisms leads to perturbation of gut homeostasis.

In the colon of *Batf2*^−/−^*Il23a*^−/−^ mice, the accumulation of T_h_1 cells was not suppressed completely, whereas numbers of T_h_17 cells and IL-17^+^ IFN-γ^+^ CD4^+^ T cells were markedly diminished. In this context, the severity of spontaneous colitis was attenuated in *Batf2*^−/−^*Il23a*^−/−^ mice. Thus, IL-17-producing CD4^+^ T cells, but not T_h_1 cells, might be linked to the pathology of intestinal inflammation attributed to BATF2 deficiency. Sema4A, a transmembrane protein, is expressed in DCs, activated T cells and T_h_1 cells (49). A previous study showed that it had non-redundant functions: DC-derived Sema4A activated T-cell priming and T-cell-derived Sema4a promoted T_h_1 differentiation (50). We previously demonstrated increased *Sema4a* expression in *Batf2*^−/−^ bone marrow-derived Mϕs stimulated with IFN-γ and LPS compared with wild-type cells (37). Thus, increased Sema4A expression in intestinal innate myeloid cells may facilitate both T-cell activation and T_h_1 differentiation in *Batf2*^−/−^ mice.

In addition to the effect on IL-17-producing CD4^+^ T cells, IL-23 induces effector functions of ILC3, which produce T_h_17-related cytokines including IL-17 and IL-22 (51). In the intestines and serum of IBD patients, elevated levels of T_h_17 signature cytokines were observed (52). In this context, IL-23-responsive ILC3 as well as T_h_17 cells were increased (22). Similar to IBD patients, the accumulation of ILC3 and T_h_17 cells was observed in the intestinal lamina propria of *Batf2*^−/−^ mice in this study, but intestinal pathology was abrogated in *Batf2*^−/−^*Rag2*^−/−^ mice, which harbor ILC3, but not T_h_17 cells. Therefore, BATF2-mediated regulation of IL-23 was essential to inhibit intestinal inflammation caused by IL-17-producing CD4^+^ T cells, but not by ILC3. IL-22 production by ILC3 in response to IL-23 is essential for host defense against pathogenic bacteria such as *C. rodentium* (39, 53), whereas ILC3 was involved in the IL-23-driven intestinal pathology during infection with *Helicobacter hepaticus* (24). In *Batf2*^−/−^ mice, the number of IL-22-producing ILCs was increased in the colon compared with wild-type mice even in the steady state. Thus, it would be interesting to identify the role of BATF2-dependent suppression of ILC3 responses in gastrointestinal infection in future studies.

Previous studies reported that spontaneous colitis was prevented in *Il10*^−/−^ mice, *Tcra*^−/−^ mice and HLA-B27 Tg rats under germ-free conditions or by microbiota deletion with antibiotics (54). In IBD patients, intestinal CD14^+^ Mϕs robustly produced pro-inflammatory cytokines including IL-23 in response to intestine-resident bacteria (7), suggesting that the inadequate activation of innate immune cells against commensal bacteria is involved in either the initiation or exacerbation of intestinal inflammation. Similar to IBD patients, alterations of microbiota composition were observed in many mouse strains with high sensitivity to intestinal inflammation (54, 55). In addition, previous studies indicated that wild-type mice engrafted with dysbiotic microbiota from spontaneous colitis model mice, such as *Nod2*^−/−^ mice (56) and TRUC mice (47, 57), developed colitis. In contrast, microbiota from CX_3_CR1^high^ Mϕ-specific *Il10ra*-deficient mice, which spontaneously develop colitis, did not induce inflammation in the colon of wild-type mice (15). Moreover, SAMP1/Yit mice, *Il2*^−/−^mice and *Rag1*^−/−^ mice transferred with Mdr1-deficient naive T cells exhibited the commensal bacteria-independent development of spontaneous intestinal inflammation (54, 58). In this study, we identified alterations in the fecal microbiota composition of *Batf2*^−/−^ mice with spontaneous colitis and ileitis, such as increased *Bacilli* and *Epsilonproteobacteria* and reduced *Erysipelotrichia*. Similar to *Batf2*^−/−^ mice, the patients with ileal CD displayed an elevated abundance of *Bacilli* (59–61). In addition, *Erysipelotrichia* was decreased in CD patients (60, 62, 63). A part of bacterial species belonging to the order *Campylobacterales* (class *Epsilonproteobacteria*) were increased in IBD patients (64–68) and implicated in the pathogenesis of IBD by promoting production of pro-inflammatory cytokines from intestinal DCs and Mϕs through activation of the NF-κB signaling (66, 68, 69). In the feces of *Batf2*^−/−^ mice, the relative abundance of *Campylobacterales* was augmented compared with wild-type mice (data not shown). Therefore, future study should characterize the effect of dysbiotic microbial communities in *Batf2*^−/−^ mice on the onset and duration of intestinal inflammation.

In this study, we focused on the function of BATF2 in gut homeostasis through the regulation of IL-23 production in myeloid cells. The IL-23 axis is thought to be a potential therapeutic target for IBD. Monoclonal antibodies against p40, which neutralize IL-12 and IL-23, provided therapeutic benefit in clinical trials of CD (5). In addition, IL-23-specific antibodies, which bind to p19 selectively, were safe and effective for the treatment of CD (5, 70). Similar to most CD patients, *Batf2*^−/−^ mice, in which IL-23 is abnormally produced in the intestinal mucosa, spontaneously developed ileitis, suggesting that BATF2 might be required for the inhibition of IL-23-mediated CD-like intestinal inflammation. IL-23 production by CD14^+^ Mϕs in CD patients (7, 71) and CD103^+^ DCs in patients with UC (72) was increased compared with normal intestines. CD4^+^ T cells co-cultured with CD14^+^ Mϕs or CD103^+^ DCs from patients with CD and UC, respectively, produced higher amounts of IL-17 and IFN-γ compared with that by those co-cultured with CD14^+^ Mϕs or CD103^+^ DCs of normal intestine (8, 71, 72). GWAS and the IBD exomes browser (https://ibd.broadinstitute.org) indicated that the missense mutations of human BATF2 bear no relation to IBD. However, the hyper-production of IL-23 by innate myeloid cells in IBD patients might be associated with dysregulated BATF2 expression possibly related to disrupted epigenetic modification of the BATF2 gene or secondary effects of IBD-related molecules such as IFNAR. Thus, it is important to analyze the expression level of BATF2 in CD14^+^ Mϕs and CD103^+^ DCs from IBD patients to gain insights for the development of novel therapeutic interventions for IBD.

## Funding

This work was supported by grants from the Ministry of Education, Culture, Sports, Science and Technology of Japan (MEXT) (grant number 501100001700), the Japan Agency for Medical Research and Development (100009619 and JP17gm1010004) (to K.T.), a Grant-in-Aid for Young Scientists (B) (MEXT) (grant numbers 26860330 and 501100001700), the Ichiro Kanehara Foundation for the Promotion of Medical Sciences and Medical Care (grant number 501100003837) and the Kurata Memorial Hitachi Science and Technology Foundation (grant number 1000010278) (to H.K.).

## Supplementary Material

dxz014_suppl_Supplementary_Figure
