# Supplementary material for: BATF2 prevents T-cell-mediated intestinal inflammation through regulation of the IL-23/IL-17 pathway
Source: Int Immunol. 2019 Feb 12;31(6):371–83. doi: 10.1093/intimm/dxz014 (PMC6528702; doi:10.1093/intimm/dxz014)
Supplement: dxz014_suppl_Supplementary_Figure [file dxz014_suppl_supplementary_figure.pdf]

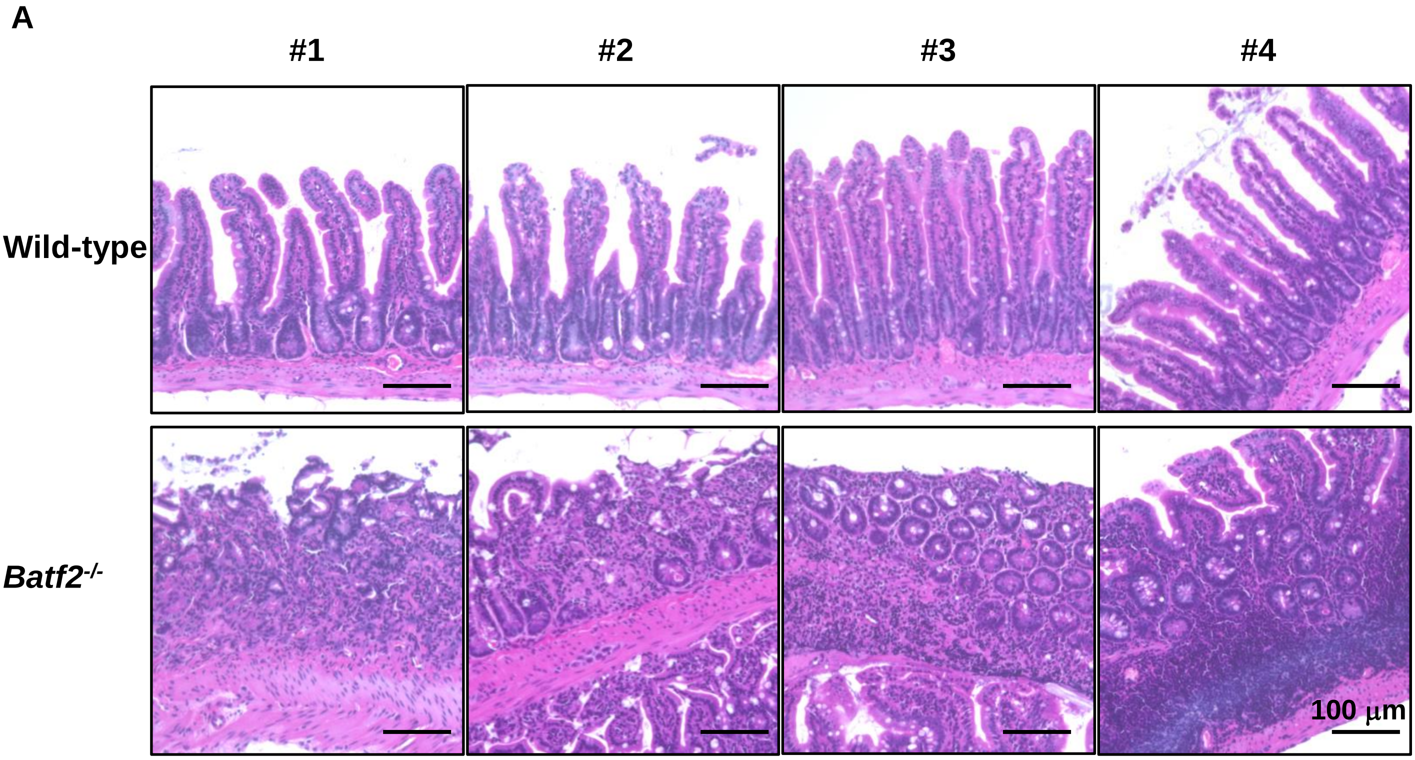

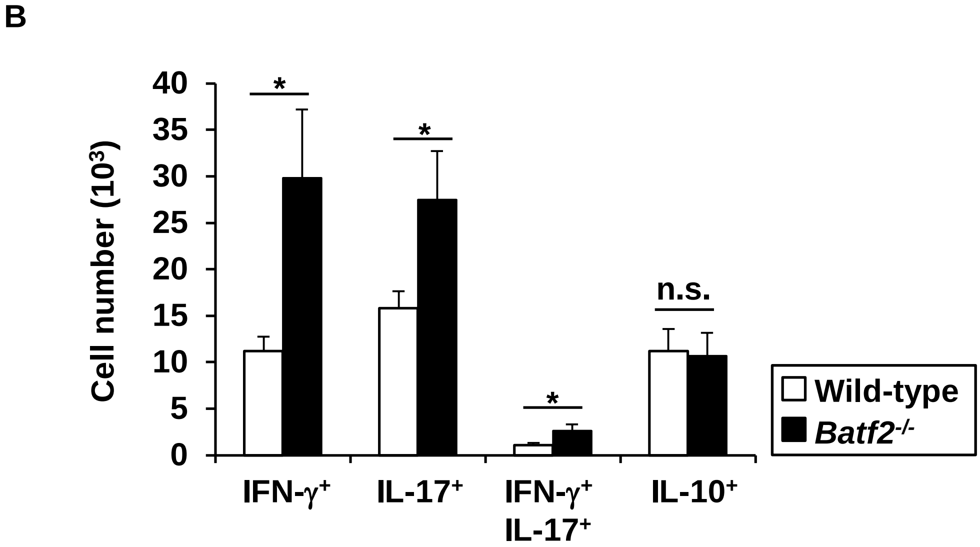

Supplementary Figure 1: *Batf2*$^{-/-}$ mice developed ileitis.
(A) Representative ileum sections of wild-type (n = 9) and *Batf2*$^{-/-}$ (n = 18) mice at the age of thirty two to forty weeks. **(B)** Number of IFN-γ-, IL-17-, and IL-10-producing CD4^{+} T cells from the small intestine of twenty four-week-old wild-type (n = 5) and *Batf2*$^{-/-}$ (n = 5) mice (mean values ± SEM). $^{*}p < 0.05$. n.s., not significant. Graphs represent data pooled from two independent experiments.
